# Supplementary material for: End Sequence Analysis Toolkit (ESAT) expands the extractable information from single-cell RNA-seq data
Source: Genome Res. 2016 Oct;26(10):1397–410. doi: 10.1101/gr.207902.116 (PMC5052061; doi:10.1101/gr.207902.116)
Supplement: Supplemental Material [file supp_gr.207902.116_Supp_Materials.tar.gz › ESAT-esat_v0.1/doc/html/interfacebroad_1_1core_1_1annotation_1_1_genomic_annotation_filter_3_01_t_01extends_01_lightweight_genomic_annotation_01_4.html]

ESAT: broad.core.annotation.GenomicAnnotationFilter< T extends LightweightGenomicAnnotation > Interface Reference


ESAT


- Main Page
- Related Pages
- Packages
- Classes
- Files

- Class List
- Class Index
- Class Hierarchy
- Class Members

All Classes Namespaces Files Functions Variables Enumerator Pages

- broad
- core
- annotation
- GenomicAnnotationFilter< T extends LightweightGenomicAnnotation >

Public Member Functions |
List of all members

broad.core.annotation.GenomicAnnotationFilter< T extends LightweightGenomicAnnotation > Interface Reference

|  |  |
| --- | --- |
| Public Member Functions | |
| boolean | accept (T annotation) |
|  | |
| boolean | isEnough (T annotation) |
|  | |

## Member Function Documentation

|  |  |  |  |  |  |
| --- | --- | --- | --- | --- | --- |
| boolean broad.core.annotation.GenomicAnnotationFilter< T extends LightweightGenomicAnnotation >.accept | ( | T | *annotation* | ) |  |

Evaluates whether current annotation meets the filtering criteria

Parameters
:   |  |  |
    | --- | --- |
    | annotation |  |

Returns
:   true if the record meets criteria

|  |  |  |  |  |  |
| --- | --- | --- | --- | --- | --- |
| boolean broad.core.annotation.GenomicAnnotationFilter< T extends LightweightGenomicAnnotation >.isEnough | ( | T | *annotation* | ) |  |

Evaluates whether annotation should stop sequencial read in of further annotations

Parameters
:   |  |  |
    | --- | --- |
    | annotaiton |  |

Returns
:   true if annotation determines that no further annotations will meet criteria.

---

The documentation for this interface was generated from the following file:

- java/broad/core/annotation/GenomicAnnotationFilter.java


---

Generated on Fri Aug 15 2014 15:10:51 for ESAT by  

 1.8.7
